# Direct structural analysis of a single acyl carrier protein domain in fatty acid synthase from the fungus *Saccharomyces cerevisiae*

**DOI:** 10.1038/s42003-024-05777-7

**Published:** 2024-01-12

**Authors:** Elnaz Khalili Samani, Amy C. Chen, Jennifer W. Lou, David L. Dai, Alexander F. A. Keszei, Guihong Tan, Charles Boone, Martin Grininger, Mohammad T. Mazhab-Jafari

**Affiliations:** 1 Department of Medical Biophysics, University of Toronto, Toronto, Canada; 2 Princess Margaret Cancer Center, University Health Network, Toronto, Canada; 3 Donnelly Centre, Toronto, Canada; 4 Department of Molecular Genetics, University of Toronto, Toronto, Canada; 5 Institute of Organic Chemistry and Chemical Biology, Buchmann Institute for Molecular Life Sciences, Goethe University, Frankfurt, Germany

**Keywords:** Enzyme mechanisms, Cryoelectron microscopy, Protein design

## Abstract

Acyl carrier protein (ACP) is the work horse of polyketide (PKS) and fatty acid synthases (FAS) and acts as a substrate shuttling domain in these mega enzymes. In fungi, FAS forms a 2.6 MDa symmetric assembly with six identical copies of FAS1 and FAS2 polypeptides. However, ACP spatial distribution is not restricted by symmetry owing to the long and flexible loops that tether the shuttling domain to its corresponding FAS2 polypeptide. This symmetry breaking has hampered experimental investigation of substrate shuttling route in fungal FAS. Here, we develop a protein engineering and expression method to isolate asymmetric fungal FAS proteins containing odd numbers of ACP domains. Electron cryomicroscopy (cryoEM) observation of the engineered complex reveals a non-uniform distribution of the substrate shuttling domain relative to its corresponding FAS2 polypeptide at 2.9 Å resolution. This work lays the methodological foundation for experimental study of ACP shuttling route in fungi.

## Abstract (web summary)

A protein engineering strategy has revealed that an acyl carrier protein domain is asymmetrically distributed within the reaction chamber of a fatty acid synthase from the yeast *Saccharomyces cerevisiae*.

## Introduction

**Fig. 1:**
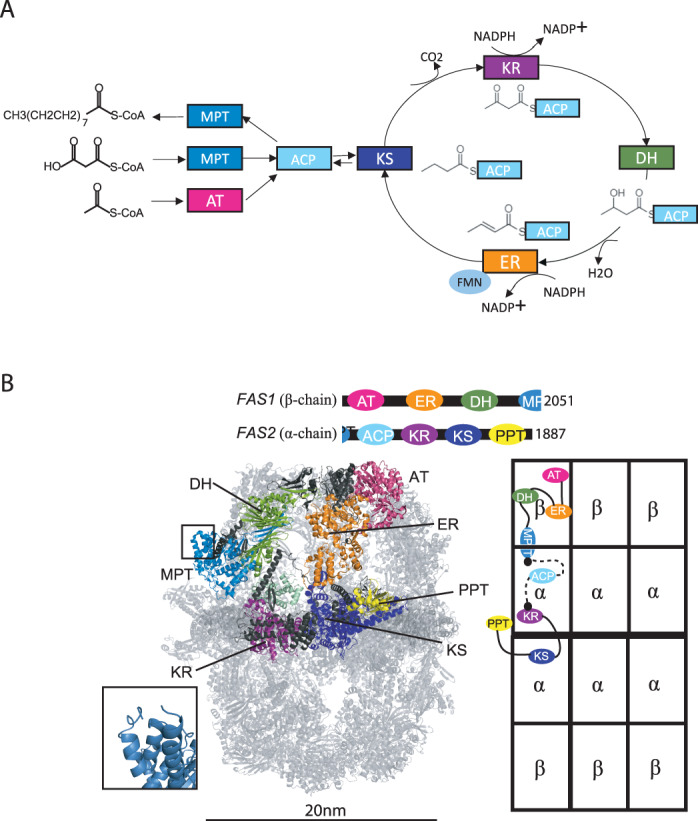
Fungal fatty acid synthesis. **A** FAS catalyzed anabolism of saturated fatty acids from acetyl- and malonyl-CoA using NADPH and FMN as cofactors. **B** Structure (PDB 6TA1) and domain organization of *S. cerevisiae* FAS. One α- and β-chain that are integrated within the MPT domain are colored in the 3D model and highlighted in the schematic architecture of FAS to the right. The N- and C-terminus of α- and β-chains, respectively, within the MPT domain are shown as an inset.

Fatty acid synthase (FAS) is responsible for de novo synthesis of palmitate (Fig. 1A), a 16-carbon chain hydrocarbon molecule involved in lipid synthesis, energy storage, and intra-cellular signaling^1–4^ Type I Fungal FAS is a well-structured and highly stable complex with approximately 95% of the assembly being static with elastic motion^4,5^. In yeast model organism, *Saccharomyces cerevisiae*, it was found that six copies of each *FAS1* and *FAS2* gene products, termed beta and alpha chains, respectively, form a 2.6 MDa barrel-shaped complex intersected at the equator with a central disk, creating two reaction chambers (Fig. 1B)^6–9^. Recently, Tma17p has been identified as a weakly interacting protein in *S. cerevisiae* FAS as an integral part of the complex and is termed γ-subunit^10^. Fatty acid synthesis including priming, elongation, and termination occurs inside the barrel by shuttling of the substrates and intermediates between catalytic domains^1,11^.

*S. cerevisiae* Acyl Carrier Protein (ACP), an 18 kDa, eight alpha-helical protein domains, is doubly tethered to the wall and central disk of the reaction chamber by a N-terminal 45 amino acid and a C-terminal 25 amino acid flexible linkers. ACP is post-translationally modified on its catalytic serine residue by attachment of a coenzyme-A derived phosphopantetheine (Ppant) moiety^12^. During fatty acid synthesis, substrate and reaction intermediates are covalently bound to the terminal thiol of Ppant enabling ACP-mediated substrate shuttling. The pivot points of the linkers are located opposite to the site of the phosphopantetheine prosthetic group, allowing the ACP to sample the entire space of the reaction chamber, as well as preventing the linkers from interfering with ACP docking to catalytic domains^13^.

ACP has been observed bound to the keto synthase (KS) domain in majority of high-resolution structures of *S. cerevisiae* FAS^6,14^. Using cryo-EM it was shown that the highest ACP occupancy is at the KS domain^15,16^, however ACP interaction landscape is prone to modulation by acyl chains^16,17^, regulatory proteins, and global conformations of the FAS scaffold^10,18^. ACP exhibits surface electrostatic complementarity to FAS catalytic domains, and it has been speculated that the ACP evolved in step with the canonical lobe to optimally interact with catalytic clefts in the reaction chamber^6,19^. Using coarse grain simulation, Anselmi et al. shown that distribution of the ACP among FAS reaction compartments is asymmetric despite the flexibility and proper length of its associated linkers^13^. The asymmetry was attributed to the molecular crowding that would interfere with the freedom of ACP achieving the electrostatic surface interactions in each of the catalytic sites equally.

The three-fold symmetry of a FAS reaction chamber and the long, flexible linkers grant one ACP domain access to three of “each” catalytic centers. For example, one ACP domain may be able to access each of the three KS catalytic sites within one reaction chamber. However, the ACP linkers are invisible in X-ray crystallography and cryoEM reconstructions because of their heterogenous conformation. In fact, ACP linkers are amongst the most divergent regions of FAS, in terms of sequence composition when compared across fungal species^7^. Since ACP linkers are structurally unresolved, it is not possible to identify the position of an ACP domain relative to its FAS2 polypeptide. Therefore, localization pattern of a single ACP domain remains elusive in fungal FAS.

Hence, an overarching question about fatty acid biosynthesis is how an ACP domain shuttles substrates and reaction intermediates among catalytic sites to undergo the iterative fatty acid biosynthesis. Lack of experimental evidence for the ACP distribution due to constraints of FAS symmetricity brought up the idea of developing a method for creating an asymmetric FAS protein that enables study of this question. Here, focusing on *S. cerevisiae* as a model system, we engineered an asymmetric fungal FAS to experimentally probe the distribution of a single ACP domain within the FAS reaction chamber.

## Results

### Protein engineering and expression setup

**Fig. 2:**
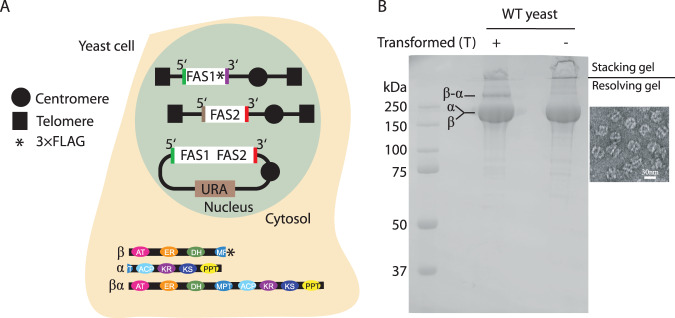
Production of asymmetric fungal FAS. **A** schematic representation for biosynthesis of asymmetric FAS proteins (i.e., prep **1**). The schematics of a yeast cells expressing asymmetric FAS protein is shown. The promoters and terminators of each gene are shown as a color-coded line flanking the respective gene. **B** SDS-PAGE analysis of purified FAS from *S. cerevisiae* yeast alone or transformed with vector expressing the β-α fusion polypeptide. A representative negative stain micrograph of FAS proteins purified from yeast transformed with the fusion FAS vector.

As a proof of concept, we set out to test if expression of a FAS1-FAS2 fusion (i.e., ^fus^FAS) polypeptide in a yeast strain expressing native *FAS1* and *FAS2* genes results in the integration of fusion polypeptide into endogenous FAS protein complex (Supplementary Table 1). A centromeric plasmid with the ^*fus*^*FAS* gene under control of FAS1 promoter and FAS2 terminator was previously designed and shown to be functional in yeast cells^20^. Gene fusion was done using *Ustilago maydis FAS* sequence that naturally expresses a fusion gene. The fusion construct lacked any affinity tag, while a 3 × FLAG was inserted at the c-terminus of the *FAS1* gene on yeast genomic DNA (W303 strain, Supplementary Table 2). A single step affinity purification against 3 × FLAG showed co-purification of a ~400 kDa polypeptide by the SDS-PAGE that approximately corresponded to the combined molecular weights of FAS1 (~220 kDa) and FAS2 (~200 kDa) polypeptides (Fig. 2). We name this protein preparation ‘prep **1**’ and assign increasing numbers for subsequent protein preparations described below. This observation indicated integration of the fusion polypeptide into the native FAS architecture. Band intensity analysis estimated an approximate 15:1 ratio of the native polypeptides (i.e., endogenous FAS1 and FAS2) to that of the ^fus^FAS polypeptide. If one fusion polypeptide is integrated per FAS assembly, one would expect a ratio of 5:1 native:fusion chain. 15:1 ratio implies that the fusion polypeptide was purified at lower stoichiometry that its native counterparts with some FAS complexes composed solely by native FAS1 and FAS2 chains.

Presence of ^fus^FAS chain at a limiting stoichiometry is beneficial for in silico alignment of the cryoEM particle images based on the position of the fusion polypeptide within the assembly as described later. However, the 15:1 ratio indicated that at least half of the purified proteins were devoid of ^fus^FAS chain. Therefore, we asked if this ratio can be modulated by a tandem affinity purification scheme with the capture of the ^fus^FAS chain followed by the capture of the native FAS1 chain. Accordingly, we modified the purification method to enable a tandem affinity capture procedure as described below.

### *Image alignment based on*^fus^FAS polypeptide

**Fig. 3:**
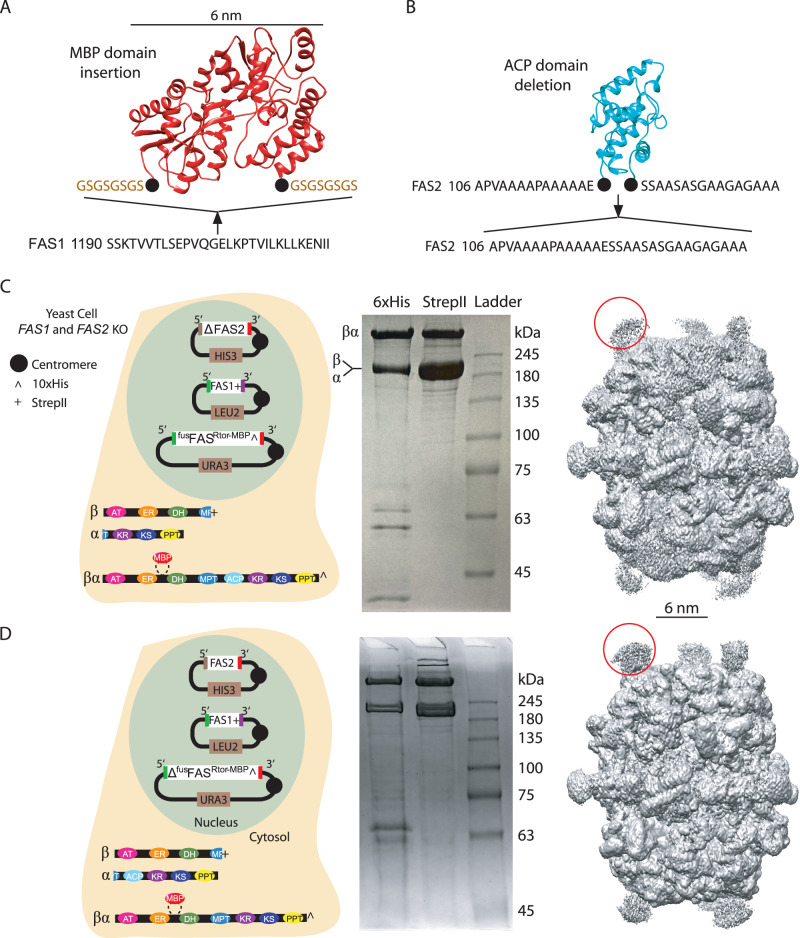
Preparation of MBP-tagged fatty acid synthase. **A** MBP insertion site in ^fus^FAS chain is shown. Two eight-residue linkers were used to doubly tether the MBP domain (PDB: 3MBP) to FAS1. **B** ACP domain deletion strategy is shown. ACP model from PDB 6TA1. Purification of asymmetric FAS with deletion of the ACP domain in (**C**) FAS2 (i.e., prep **4**) and **D**) ^fus^FAS chain (i.e., prep **5**). From left to right: schematic of cell expressing the MBP tagged constructs, SDS-PAGE of tandem affinity purification (Ni-NTA pull down followed by twin strep-II tag affinity purification) of the engineered FAS, and 3D cryoEM density maps of the purified complex. One MBP domain is highlighted with red circle in each cryoEM map.

To facilitate FAS particle image alignment based on the position of the ^fus^FAS chain; a unique structural feature is needed that distinguishes the engineered polypeptide from its native counterparts. We chose maltose-binding protein (MBP), which is a 6 nm wide soluble tag and considered insertion at two distinct sites on the ^fus^FAS chain. These sites included single tethering to the N-terminus of ^fus^FAS chain (i.e., ^fus^FAS^N-MBP^ chain) and double tethering to a loop in the FAS1 segment of the ^fus^FAS chain that is naturally disconnected in FAS from *Rhodosporidium toruloides* yeast^21^ (i.e., ^fus^FAS^Rtor-MBP^ chain). Ideally, insertion of the MBP tag should have minimal impact on FAS structural assembly. Therefore, we decided not to insert the MBP domain at the natural breaking point in *S. cerevisiae* FAS since MPT domain is the site of co-translational assembly of FAS1 and FAS2 polypeptides^22^. MBP domain was linked to the N-terminus of the ^fus^FAS chain with a two amino acid linker sequence (i.e., GS) to minimize domain flexibility that can degrade the accuracy of image classification based on MBP occupancy. Similarly, to minimize MBP domain flexibility, an eight amino acid linker (i.e., GSGSGSGS) was used for MBP double tethering (Fig. 3A) based on the distance between the N- and C-terminal ends of the MBP domain (i.e., 36 Å based on PDB 3MBP^23^).

For the two MBP insertion sites discussed above, impact of structural modification on FAS assembly was assessed in a yeast strain with *FAS1* and *FAS2* genes deleted (strain BY.PK1238, Supplementary Table 2). We hypothesized that expression of the fusion polypeptide in the absence of native chains will result in FAS assemblies composed completely of engineered chains (i.e., preps **2** and **3**). All constructs were expressed by transformation of the yeast cells with a centromeric plasmid containing the target MBP-tagged FAS fusion gene with a C-terminal 10×HIS affinity tag. Prep **2** (i.e., 6 × ^fus^FAS^N-MBP^ chains) was expressed and purified but was unstable as judged by lack of image alignment for the apical region of the FAS dome (Supplementary Fig. 1A, B). The observed structural instability is likely due to the proximity of the N-termini of ^fus^FAS chains (<10 Å) at the 3-fold axis of the barrel-shaped structure that results in steric clashes between the MBP tags. Surprisingly, however, the *FAS1* and *FAS2* KO yeast cells were able to grow in the absence of exogenous fatty acids by transformation with centromeric plasmid expressing ^fus^FAS^N-MBP^ chain, indicating that the complex maintained minimal catalytic activity necessary for the fungal growth. Prep **3** (i.e., 6 × ^fus^FAS^Rtor-MBP^ chain) was not dependent on exogenous fatty acids as well and resulted in 2D classes and a 3D cryoEM map representative for the native FAS assemblies (Supplementary Fig. 2). The position of the MBP density was clearly observed at the site of insertion in the cryoEM density map. Rigid body fitting of FAS1 and FAS2 chains from native FAS atomic model (PDB 6TA1)^24^ into the cryoEM density demonstrated minimal structural impact from the inserted MBP domain (Supplementary Fig. 2C). ^fus^FAS^Rtor-MBP^ chain was therefore chosen to be co-expressed with native FAS1 and FAS2 polypeptides to generate asymmetric FAS assemblies containing MBP domains as discussed below.

The centromeric plasmid expressing ^fus^FAS^Rtor-MBP^ chain was co-transformed in a *FAS1* and *FAS2* KO yeast strain with centromeric plasmids containing native *FAS1* and ^Δ^*FAS2* genes, with ^Δ^ representing ACP deletion (prep **4**). To delete ACP domain, the N- and C-terminal linkers of the shuttling domain were fused at their junction with the shuttling domain (Fig. 3B). *FAS1* gene was tagged with a C-terminal twin strep-II tag. Tandem purification of FAS from this strain estimated a chain intensity ratio of approximately 5:2 of native:fusion polypeptide (Fig. 3C). Mixed chain FAS was also purified even when ACP domain was deleted on the fusion chain, while maintained on the FAS2 chain (i.e., prep **5**) (Fig. 3D). Applying 2D classification and 3D reconstruction, we obtained cryoEM maps that closely resembled the structure of the wild type FAS, except for weak densities corresponding to the MBP domains that protruded out of the barrel (Fig. 3C, D). MBP densities are considerably weaker than the corresponding densities for the FAS barrel since most FAS complexes have only one MBP tagged fusion chain and image alignment is primarily influenced by the FAS barrel protein that compose ~95% of the signal (i.e., protein mass) in the particle images.

**Fig. 4:**
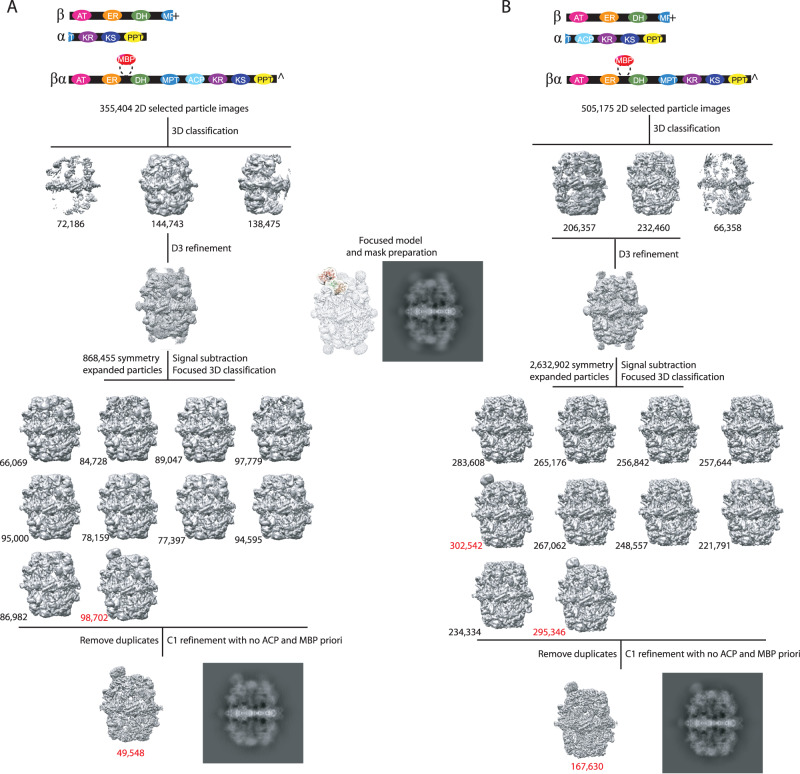
In silico purification and alignment of FAS complexes containing one MBP tag. Polypeptides composing each sample in panels (**A**) from prep **4** and (**B**) prep **5** are shown on the top. Transparent cryoEM map in the middle, shows the region of the complex used in focused 3D classification. 2D projections of selected 3D cryoEM density maps are shown. All 3D maps and 2D projections are shown from the same viewing direction.

We then set out to align FAS particle images based on the position of the MBP fusion chain from prep **4** and **5** (Fig. 4A, B, respectively). 2D and 3D classifications were used to select intact particle projections and discard partially unfolded particle images that are the result of FAS interaction with air-water interface as shown previously via electron tomography studies on fungal FAS^25^. Intact particle images were symmetry expanded based on alignment information from a consensus 3D refinement with D3 symmetry imposed. A focused mask composed of segments of ER and DH domains (i.e., FAS1 residues 561-812, 1056-1109, 1123-1256), plus the expected position of the MBP domain in prep **4** and **5** was made. The expected position of MBP domain was determined from the cryoEM map of prep **3** (Supplementary Fig. 2). Symmetry expanded particles were 3D classified using orientation information from the consensus D3 refinement. We previously used a similar strategy to classify FAS particles based on ACP domain occupancy at the dehydratase catalytic site in fungal FAS^17^. Approximately 10% and 22% of the total symmetry expanded particle images classified in 3D classes with a strong signal corresponding to the MBP domain, for prep **4** and **5**, respectively. We then removed duplicate particles to revert to original non symmetry expanded particle images. Duplicate particles indicate that the symmetry rotated and translated version of the same particle image was classified as containing MBP domain, which is indicative of FAS assemblies that have more than one ^fus^FAS^Rtor-MBP^ chain. Therefore, by removing duplicates, assemblies with only one ^fus^FAS^Rtor-MBP^ chain were selected. 34% and 38% of the intact (i.e., not disassembled by air-water interface) FAS particle projections from prep **4** and **5**, respectively, contained a single fusion chain with the remainder containing more than one. Intact FAS projections containing a single MBP domain were used to reconstruct a 3D density map with no symmetry imposed starting from the alignment information from their respective focused 3D classification (Supplementary Fig. 3). Reference volume for the final 3D reconstruction did not contain any signal for MBP or ACP domains and orientation search was restricted to 1 degree rotational and 1 Å translational searches. This was to ensure that MBP and ACP density reconstruction in the final map were not affected by priori bias and no major deviation from the orientation assignment from the 3D classification occurs during C1 refinement since 95% of signal is contributed by FAS barrel.

**Fig. 5:**
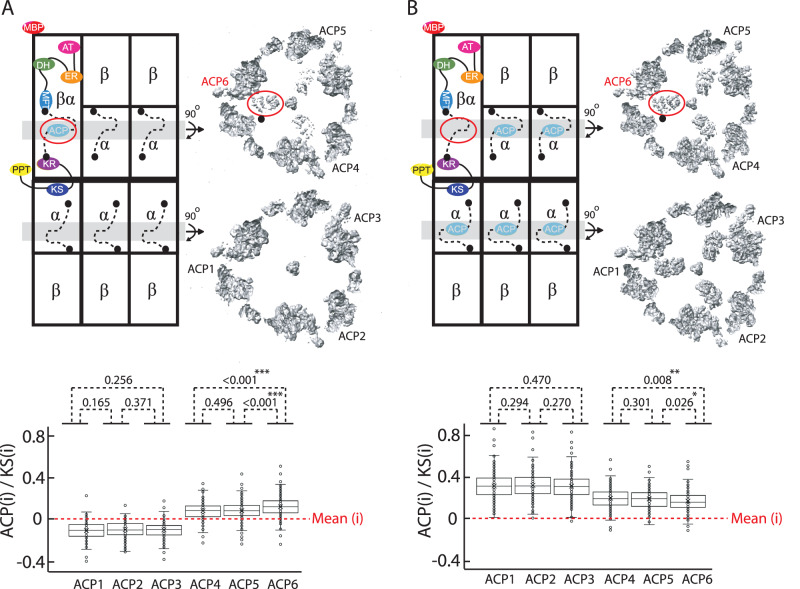
ACP domain distribution in asymmetric *S. cerevisiae* FAS. Schematic illustration and slices of the cryoEM density maps of FAS complexes containing (**A**) one (i.e., prep **4**) and (**B**) five ACP domains (i.e., prep **5**). The tethering point of ACP to the MPT domain of ^fus^FAS chain is highlighted with a black dot in the cryoEM slices. cryoEM density (denoted as (i)) around each amino acid of each ACP domain is normalized against the cryoEM density of KS domain (Supplementary Fig. 4) and is plotted as box plots. Mean voxel density is set to zero for each 3D reconstruction. See material and method for details of density quantification. Final cryoEM density reconstruction (Table 1, EMD-40784 and EMD-40785) was used for density quantification of FAS2 amino acids 141 to 166 corresponding to the ACP domain (*n* = 161). Differences are statistically assess using a homoscedastic, one-tailed *T* test.

MBP domain is localized strongly to only one asymmetric unit of FAS in the final cryoEM reconstruction from prep **4** (Fig. 4A) and ACP densities are almost non-existent in the reaction chamber opposite to the one containing the MBP domain (Fig. 5A). Densities corresponding to the ACP domain within the MBP-containing reaction chamber are non-uniformly distributed in each asymmetric unit with a small but statistically significant increase in density for the ACP domain closest to the MBP-fusion chain (Fig. 5A). Therefore, one ACP domain can reach all three ketoacyl synthase sites within a reaction chamber with a small preference for localization closer to its N-terminal tethering point attached to the MPT domain of own protomer (Fig. 5A, black dot on cryoEM slice).

Steric occlusions by other ACP domains may influence the localization of the substrate shuttling domain^13,17^. Therefore, to further test our observations in FAS from prep **4** in a scenario where there are two ACP domains within a reaction chamber containing MBP domain, we analyzed the FAS complex where ACP was present in the native FAS2 chain but absent in the MBP-fusion chain (i.e., prep **5**). Co-expression of FAS1 and FAS2 chains with ^Δfus^FAS^Rtor-MBP^ enabled purification of assembly **5** as discussed above. Analysis of cryoEM densities corresponding to the shuttling domains, demonstrated ACP density for all three ketoacyl synthase sites in the MBP-containing reaction chamber but this time with weaker ACP density in proximity of the MBP-fusion chain (Fig. 5B), corroborating the observations for assembly **4**. Overall, our studies on asymmetric FAS support the notion of an ACP domain ability to interact with all three KS catalytic sites within one reaction chamber with partial preference to interact with a KS domain that is closer to the MPT tether of ACP’s own protomer.

## Discussion

One of the key questions in the FAS biosynthesis mechanism relates to the ACP domain interaction landscape within a reaction chamber. This is a multifaceted question since a multitude of factors can impact ACP localization^26^, including the nature of the acyl-moiety bound to the terminal thiol of Ppant arm and relative position of catalytic domains to the tethering points of ACP linkers on the reaction chamber. In the conformationally dynamic mammalian FAS^27–29^, the ACP may in addition be restricted in its trajectory by the positional variability of domain, but such constraints do not apply for the rigid fungal FAS. When FAS is purified from yeast, the multienzyme complex loses access to substrates and NADPH and therefore ACP domain will be in a heterogenous chemical state with different acyl intermediates conjugated to its Ppant arms. Considering the above and the time scale of protein domain dynamics that ranges from nano- to micro-seconds, our observation here is a time and chemical average of ACP distribution in FAS when it is isolated from yeast. It is intriguing to observe that one ACP domain can interact with all three available ketoacyl synthase sites in the reaction chamber and populate those sites. This observation is consistent with the perception that an ACP domain cannot just reach the closest set of active sites but also serve more distant catalytic sites.

Although ACP domains show a stochastic interaction pattern at the KS domains, our study indicates that FAS2 chains tend to keep their respective shuttling domain in close proximity to their anchoring points within the *S. cerevisiae* multienzyme complex. Our study indicates that the strongest ACP density belongs to a KS site where both the N- and C-terminus of the ACP are closest to the tethering points of its own FAS2 chain. Tethering point of the N-terminus of an ACP to the MPT domain is 3.8 (own FAS2 chain), 7.1, and 7.3 nm away and the distance of the C-terminal tethering point of the ACP to the FAS2 tethering point is 2.3 nm (own FAS2), 3.2 nm and 3.2 nm. These observations suggest that proximity of the ACP domain tethering point impacts localization of the substrate shuttling domain in fungal FAS. In addition, the length of the linkers as well as their persistence length^30^, the latter defined by the amino acid composition, will define the distribution of ACPs, and the knowledge of these properties allow predicting ACP localization in these enzymes. As an example, the ACP domains of type I FAS from *Mycobacterium tuberculosis* are shorter^7^ compared to those from *S. cerevisiae*, which could imply a more proximal shuttling pathway of the ACP domains relative to its tethering points in *M. tuberculosis*.

A caveat in our study is the deletion of one or two ACP domains. Simulation studies have shown that ACP linkers play a major role in the distribution of the shuttling domain through steric collisions and therefore were present in our deletion constructs. However, ACP domain themselves were also shown through simulation to provide some steric guidance in determining the interaction landscape of the shuttling domain^13^. Therefore, it is possible that our observations here are impacted by more space available for ACP movements in both assemblies **4** and **5**.

To experimentally study translocation of a single ACP domain in a chemically controlled manner and in the presence of all three ACP domains within a reaction chamber, one can envision a similar protein production platform with following modification. Instead of domain deletion, ACP domain can be inactivated via a point mutation of S180 (*S. cerevisiae* numbering) to alanine in the FAS2 polypeptide while maintain wild type ACP on the MBP fusion polypeptide. We have shown previously that ACP can be translocated to and away from KS domain in *S. cerevisiae* FAS by stalling the FAS reaction cycle using inactivating point mutation on a target catalytic site (e.g., DH or ER domains)^17^. Similar ACP translocation strategy on FAS complexes with holo ACP domain on the MBP-fusion chain will enable tracking of the shuttling domain to the stalled catalytic sites via cryoEM. Therefore, our work presented here provide an experimental foundation to probe a single ACP domain localization in fungal FAS.

## Methods

### Cloning

Plasmid constructs used in this study are listed in Supplementary Table 3. The plasmid containing the chimeric *FAS1-FAS2* fusion (fusFAS plasmid), was constructed as described^20^. Series of overlap extension PCR2 performed for substitution of HIS and URA auxotrophic markers along with the insertion of the 10× Histidine affinity tag at the C-terminus of the fused *FAS1-FAS2* gene.

For the insertion of Maltose Binding Protein (MBP) to the N-terminus of the beta chain in the fused *FAS1-FAS2* construct, the MBP was amplified from pET15b -MBP plasmid with primers that contained 50 base pair homology with the *FAS1* linked by a single Glycine to the 18 base pair homology with the C-terminus of *malE* gene. This PCR fragment was then elongated to contain restriction digest sites and subsequently cloned to the NheI-PacI double-digested fused *FAS1-FAS2* using T4-ligation.

For insertion of MBP to the conserved break of Rhodosporidium toruloids (Rtor) fatty acid synthase into the fused *FAS1-FAS2*, MBP elongation was performed out of pET15b-MPB plasmid using primers that contained a homologous region of the MBP linked by 8 repeats of Glycine-Serine to the homologous region of *FAS1* or *FAS2*. This fragment was then elongated from both sides in a way to contain restriction digestion sites and then cloned to the NheI-PacI doubled digested fused *FAS1-FAS2* using NEBuilder (Gibson assembly).

For deletion of the ACP from the FAS2 and ^fus^FAS^Rtor-MBP^, a primer was designed containing a sequence immediately before and after the ACP linker’s sequence. This primer was used along with a second primer to amplify a fragment from the centromeric plasmid, which contained restriction digested sites of NheI and PacI. The resulting PCR fragment was double-digested with NheI-PacI and then cloned into the digested plasmid using T4-ligation. Sequences were verified using full plasmid sequencing.

### Yeast strain

Two yeast strains used in this study are listed in Supplementary Table 2. The haploid *S. cerevisiae* strain W303-FAS1-3×FLAG was prepared as previously reported^16^. The *FAS1 FAS2* double knockout *S. cerevisiae* strain with BY background (BY.PK1238_FAS1-FAS2-dKO) was created by replacing each open reading frame for *FAS1* and *FAS2* with the KanMX cassette^31^, and is selected for by growth in presence of 200–500 μg/mL G418 antibiotic.

### Transformation and expression

The fused *FAS1-FAS2* expression vector was transformed into W303-FAS1-3xFLAG yeast strain and selected using HIS3 nutritional marker. Each of the MBP fusion expression vectors were either transfected alone or co-transformed with MF639K1_PRS315_FAS1 (C-terminus twin strepII tag) and MF319d_PRS313_FAS2 to the BY.PK1238_FAS1-FAS2-dKO strain. The transformation was performed firstly by growing each strain on the nutrient-rich plates (Yeast Extract Peptone Dextrose) with G418 (200 μg /mL) and subsequent growth in the same media at 30 degrees to the OD_660_ = 0.6–1. Transformation into yeast cells were via the standard lithium acetate method followed by selection on SD plates made up of synthetic dropout media (selective for each expression plasmid’s auxotrophic marker) and D-Glucose. Transfected colonies were then re-streaked on new selective plates and grown for additional two days at 30 degrees.

### Protein purification

To purify fused MBP-tagged FAS constructs from *S. cerevisiae* (i.e., prep **2** and **3**), transfected cells were grown in selective media followed by scale-up in 1 Litter YPD to OD_660_ = 2.5-3. Cells were harvested via centrifugation at 4000 g, 4 °C, 15 min and resuspended in lysis buffer (pH 7.5, 33.5 mM KH_2_PO_4_, 66 mM K_2_HPO_4_, 300 mM KCl, 10 mM Imidazole) with protease inhibitors (0.5 mM PMSF, 1 mM benzamidine, 5 mM aminocaproic acid, 10 mM NaF, 50 mM β-glycerophosphate). Cell lysis was performed by bead-beating in the Stainless-Steel Chamber Jar (Biospec Products) with 0.5 mm glass beads for 10 cycles of 30 s bead beating followed by 1 min rest to cool the sample. The resultant lysate was spun twice, first at 4000 g at 4 °C for 15 min to eliminate crude cell debris and then at 110,000 g at 4 °C for 1 h to remove fine debris. The lysate was filtered through a 0.22 μm syringe filter. For prep **1**, the lysate passed through the Poly-Prep Chromatography Column (Bio-Rad, United States) with 0.5 ml bed volume of Anti-FLAG M2 affinity gel (Sigma-Aldrich, United States). Following protein binding, the column was washed with 5 bed volumes of lysis buffer, followed by 5 bed volumes of Tris Buffered Saline (TBS) (pH 7.4, 50 mM Tris, 150 mM NaCl). Protein was eluted with 3 bed volumes of 150 μg/mL 3 × FLAG peptide in TBS buffer. For preps **2** and **3**, Ni- NTA affinity chromatography (20 mM imidazole wash, 300 mM imidazole elution) followed by size-exclusion (Superose 6 Increase 10/300 GL column in TBS buffer) was used to purify the fused FAS1-FAS2, MBP-tagged fatty acid constructs.

For the tandem purification of Rtor-MBP tagged ^fus^FAS and the endogenous FAS (i.e., preps **4** and **5**), first Ni-NTA affinity beads were used to purify the Rtor-MBP tagged FAS which had a 10× Histidine, followed by passing through Streptactin column connected to ÄKTA pure to capture endogenous FAS with the twin strep-II tag on C-terminus of FAS1 and eluting with 2.5 millimolar desthiobiotin.

### Negative stain electron microscopy

For preparing negative stain grids, Cu/Rh grids coated with an amorphous carbon support layer were glow discharged for 25 sec using 15 mA current, in air at a pressure of 0.39 bar. Proteins (0.01–0.05 mg/ml) were dispersed on the grid and incubated for 2 min at room temperature. Excess proteins were washed three time in 50 μl water drops followed by staining with a 50 μl of 0.02% w/v Uranyl Acetate. Grids were screened on the Talos L120C equipped with an LaB_6_ filament and a Ceta-M camera at 57,000 × (2.48 Å/px) and 73,000 × (1.94 Å/px) with ~50 e^−^/Å^2^ dose. Image analysis for the N-terminus MBP-fused FAS1-FAS2 was performed by *cryoSPARC v3.2* on a small dataset of 25 micrographs. CTF estimation was performed with CTFFIND4. Particles were picked manually to generate templates for automatic particle picking using template matching, followed by iterations of 2D classification to isolate the best particles.

### Cryo-EM specimen preparation

Purified FAS complexes concentrated to 2 mg/ml followed by applying 3 μl onto in-house nanofabricated holy sputtered gold girds with a hole size of ~2 μm and a 4 μm period. Grid freezing was done in Vitrobot Mark IV (FEI) with 3-second blotting at 4 °C, 90% humidity and using liquid ethane kept at liquid nitrogen temperature.

### Cryo-EM data collection and analysis

Image processing statistics can be found in Table 1. Cryo screening was done using a ThermoFisher Scientific (TFS) Talos L120C G2 transmission electron microscope (TEM) equipped with a LaB_6_ crystal and a Ceta camera, operating at 120 kV Cryo-EM. Movies for high-resolution analysis were collected with a Titan Krios G3 microscope operated at 300 kV and equipped with Falcon 4i camera (TFS). Automated data collection was performed by EPU.

**Table 1:** cryoEM Data collection and refinement statistics.

	^fus^FAS^Rtor-MBP^ (EMD-40783)	^fus^FAS^Rtor-MBP^ + FAS1 + FAS2^ΔACP^ (EMD-40784)	^ΔACP-fus^FAS^Rtor-MBP^ + FAS1 + FAS2 (EMD-40785)
**Data collection and processing**			
Magnification	75,000	75,000	75,000
Voltage (kV)	300	300	300
Electron exposure (e–/Å^2^)	54.76	40.36	36.00
Defocus range (μm)	0.6–2.5	0.6–2.5	0.6–2.5
Pixel size (Å)	1.03	1.03	1.03
Symmetry imposed	C1	C1	C1
Initial particle images (no.)	102,657	623,888	640,558
Final particle images (no.)	71,932	49,548	167,630
Map resolution (Å) (FSC 0.143)	3.21	3.19	2.87
Map resolution range (Å)	10–2.5	10–2.5	10–2.5
**Refinement**			
Initial model used (PDB code)	N/A	N/A	N/A
Model resolution (Å)	N/A	N/A	N/A
FSC threshold			
Model resolution range (Å)	N/A	N/A	N/A
Map sharpening *B* factor (Å^2^)	N/A	N/A	N/A
*Model composition*	N/A	N/A	N/A
Non-hydrogen atoms			
Protein residues			
Ligands			
*B factors (Å*^*2*^*)*	N/A	N/A	N/A
Protein			
Ligand			
*R.m.s. deviations*	N/A	N/A	N/A
Bond lengths (Å)			
Bond angles (°)			
*Validation*	N/A	N/A	N/A
MolProbity score			
Clashscore			
Poor rotamers (%)			
*Ramachandran plot*	N/A	N/A	N/A
Favored (%)			
Allowed (%)			
Disallowed (%)			

Patch motion correction and patch CTF estimation were used to align and averaged movie frames and correct for CTF in *cryoSPARCv4* with last movie frame ignored. Automatic particle picking was done using template matching with template generated via manual particle image picking and 2D classification. For all constructs discussed in this study, the initial auto-picked particle image stacks were subjected to 2D classifications and heterogenous refinements to remove images corresponding to contaminants and broken particles. The final particle image stacks were subjected to local and global CTF refinements followed by a homogenous refinement with either no symmetry or D3 symmetry enforced. Default parameters were used unless stated otherwise. For reconstruction of asymmetric FAS cryoEM density maps, D3 refined particle images stacks were symmetry expanded and were 3D classified without orientation search using a focused mask as described in the manuscript. 3D classifications without orientation search were done in *cryoSPARC v4.1.0* using default parameters. Particle images belonging to the MBP-containing 3D classes were selected followed by the removal of duplicate images. These images were used to reconstruct the final 3D volume of the asymmetric FAS constructs as described in the manuscript. More specifically, default parameters were used in the final local refinement except for (1) rotation and shift search extends were limited to 1 degree and 1 Å, respectively, (2) maximum alignment resolution was set to 0.1 degrees, (3) force-redo GS split was set to on, and 4) non-uniform refine enable was set to off.

cryoEM densities corresponding to ACP domains (residues 141-302 of FAS2) docked at the KS binding sites were quantified in UCSF Chimera v1.16 for each 3D reconstruction of asymmetric FAS from preps **4** and **5**. Specifically, atomic model of FAS (PDB 6TA1) was rigid body docked into the respective cryoEM maps of the asymmetric FAS aligned based on MBP domain as described above. Densities around each amino acid of each ACP domain was then measured at an identical threshold value within each map. These densities were normalized to the average cryoEM density of KS heterodimer composed of two sets of residues 1120-1179 contributed from two FAS2 chains (Supplementary Fig. 4) for each respective cryoEM map. This region of the KS heterodimer faces both reaction chambers of FAS and forms two docking sites on each side of the FAS central disks for ACP binding in each respective reaction chamber. Data were plotted as box plots in excel indicating median line, first and third quartiles, whiskers, and outliers (Fig. 5).

### Statistics and reproducibility

CryoEM datasets contain hundreds of thousands to millions of particle images, creating an inherent replication in the method. However, the extensive cryoEM dataset could not be duplicated due to the expenses associated with electron microscope data collection and the constraints of available disk space. The size of the cryoEM data was chosen based on its capacity to achieve a final 3D reconstruction with a global resolution of 3.5 Angstroms or better. Statistical analysis for the differences in cryoEM densities of amino acids corresponding to the ACP domains of FAS in Fig. 5 was done using homoscedastic, one-tailed *T* tests.

### Reporting summary

Further information on research design is available in the Nature Portfolio Reporting Summary linked to this article.

### Supplementary information

Supplementary Information

Description of Additional Supplementary Files

Supplementary Data 1

Supplementary Data 2

Reporting Summary

## Data Availability

cryoEM density maps are deposited into EMDB with accession codes EMD-40783, EMD-40784, EMD-40785. Source data for Fig. 5 can be found in supplementary data 1 and supplementary data 2. Uncropped and unedited images of gel shown in Figs. 2 and 3 are provided in supplementary Fig. 5. All materials are available upon reasonable request.
