# Supplementary material for: Direct structural analysis of a single acyl carrier protein domain in fatty acid synthase from the fungus Saccharomyces cerevisiae
Source: Commun Biol. 2024 Jan 12;7:92. doi: 10.1038/s42003-024-05777-7 (PMC10786820; doi:10.1038/s42003-024-05777-7)
Supplement: Supplementary file 1 — Supplementary Information [file 42003_2024_5777_MOESM1_ESM.pdf]

Direct Structural Analysis of a Single Acyl Carrier Protein Domain in Fatty Acid Synthase from the Fungus *Saccharomyces cerevisiae*

Elnaz Khalili Samani[1], Amy C. Chen[1], Jennifer W. Lou[1], David L. Dai[1], Alexander F.A. Keszei[1,2], Guihong Tan[3], Charles Boone[3,4], Martin Grininger[5], Mohammad T. Mazhab-Jafari[1,2]*

[1]Department of Medical Biophysics, University of Toronto,
[2]Princess Margaret Cancer Center, University Health Network, Toronto, Ontario, Canada.
[3]Donnelly Centre, Toronto, ON, Canada.
[4]Department of Molecular Genetics, University of Toronto, Toronto, ON, Canada.
[5]Institute of Organic Chemistry and Chemical Biology, Buchmann Institute for Molecular Life Sciences, Goethe University, Frankfurt, Germany.

*Correspondence: mohammad.mazhabjafari@utoronto.ca

Supplementary Information:

Tables…………………………………………………………………… pages 2-4

Figures……………………………………………………………………pages 5-9

Supplementary References……………………………………………….page 10

Supplementary tables:

Supplementary Table 1. Overview of five protein preparations used in this study.

Protein Prep No.	Transformation Plasmid	Expression Yeast Strain	Purification Method	Polypeptides of Purified Protein	Hypothesis Tested (True or False)
Prep 1	Fused *FAS1-FAS2* [a]	W303-FAS1-3xFLAG[b]	anti-flag affinity liquid chromatography and size exclusion chromatography	Mixture of fused and endogenous FAS polypeptides	A fused FAS1-FAS2 heteropolymer can be integrated into FAS protein assembly composed of native FAS1 and FAS2 polypeptides.
Prep 2	MBP-fused*FAS1-FAS2*-10xHis (N-terminus MBP tag)	BY.PK1238_FAS1-FAS2-dKO[c]	Ni- NTA affinity liquid chromatography followed by size exclusion chromatography	Fused FAS1-FAS2 polypeptide with a MBP tag at the N-terminus of Beta chain	FAS protein assembly can be tagged with MBP domain at the N-terminus of FAS1-FAS2 heteropolymer with minimal structural perturbation.
Prep 3	Rtor-MBP-fused-*FAS1-FAS2*-10xHis	BY.PK1238_FAS1-FAS2-dKO[c]	Ni- NTA affinity liquid chromatography followed by size exclusion chromatography	Fused FAS1-FAS2 polypeptide with the MBP tag at the gene splitting site of the Beta chain	FAS protein assembly can be tagged with MBP domain between the ER and DH domains of FAS1-FAS2 heteropolymer with minimal structural perturbation.
Prep 4	Rtor-MBP-fused-*FAS1-FAS2*-10xHis MF639K1_*FAS1*-StrepII MF319d_FAS2-ΔACP	BY.PK1238_FAS1-FAS2-dKO[c]	Ni- NTA affinity liquid chromatography Followed by strep-tactin liquid chromatography	Fused FAS1-FAS2 protein with the MBP tag at the gene splitting site of the Beta chain mixed with endogenous FAS	Distribution of an ACP domain can be determined from a FAS protein with mixed polypeptides containing native FAS1 and FAS2$^{(\Delta ACP)}$ and MBP-tagged FAS1-FAS2 heteropolymer.
Prep 5	Rtor-MBP-fused-*FAS1-FAS2*-10xHisΔACP MF639K1_*FAS1*-StrepII MF319d_FAS2	BY.PK1238_FAS1-FAS2-dKO[c]	Ni- NTA affinity liquid chromatography Followed by strep-tactin liquid chromatography	Fused FAS1-FAS2 protein with the MBP tag at the gene splitting site of the Beta chain mixed with endogenous FAS	Distribution of five ACP domains can be determined from a FAS protein with mixed polypeptides containing native FAS1 and FAS2, and MBP-tagged FAS1-FAS2$^{(\Delta ACP)}$ heteropolymer.

a from[1]
b from[2]
c from[3]

Supplementary Table 2. *S. cerevisiae* strains used in this study as expression system.

Name	Description	Genotype
W303-FAS1-3xFLAG[a]	3xFLAG cloned at *FAS1* C-terminus	MATa/MATα {leu2-3,112 trp1-1 can1-100 ura3-1 ade2-1 his3-11,15} [phi$^+$] FAS1::3x Flag-URA3
BY.PK1238_FAS1-FAS2-dKO[b]	FAS1 and FAS2 reading frames replaced with KanMX cassette	Mata; his3Δ0 leu2Δ0 ura3Δ0 TRP1 lys2D0 MET15 fas1::uptag-kanMX4-downtag fas2::uptag-kanMX4-downtag

a From [2]
b From [3]

Supplementary Table 3. Plasmid constructs used in this study.

Name	Background	Description
Fused *FAS1-FAS2*[a]	pRS313	*FAS1-FAS2* fusion, AmpR resistance, Histidine auxotroph
MBP-fused*FAS1-FAS2*-10xHis	pRS313	*FAS1-FAS2* fusion gene with N-terminus MBP tag and C-terminus 10xHis tag, AmpR resistant, Uracil auxotroph
Rtor-MBP-fused-*FAS1-FAS2*-10xHis	pRS313	*FAS1-FAS2* fusion gene with MBP inserted in interface between R. toruloides *FAS1* and *FAS2* genes and 8 aa linker, AmpR resistant, Uracil auxotroph
Rtor-MBPΔACP-fused*FAS1-FAS2*-10xHis	pRS313	*FAS1-FAS2* fusion gene with MBP inserted in interface between R. toruloides *FAS1* and *FAS2* genes and 8 aa linker and ACP deleted, AmpR resistant, Uracil auxotroph
MF319d_*FAS2*	pRS313	*S. cerevisiae* FAS2 gene, HIS3 marker

MF639K1_*FAS1*-StrepII	pRS313	*S. cerevisiae* FAS1 gene with C-terminus strepII tag, LEU2 marker
MF319d_FAS2-ΔACP	pRS313	*S. cerevisiae* FAS2 gene, HIS3 marker with ACP deleted

a From [1]

Supplementary Figures:

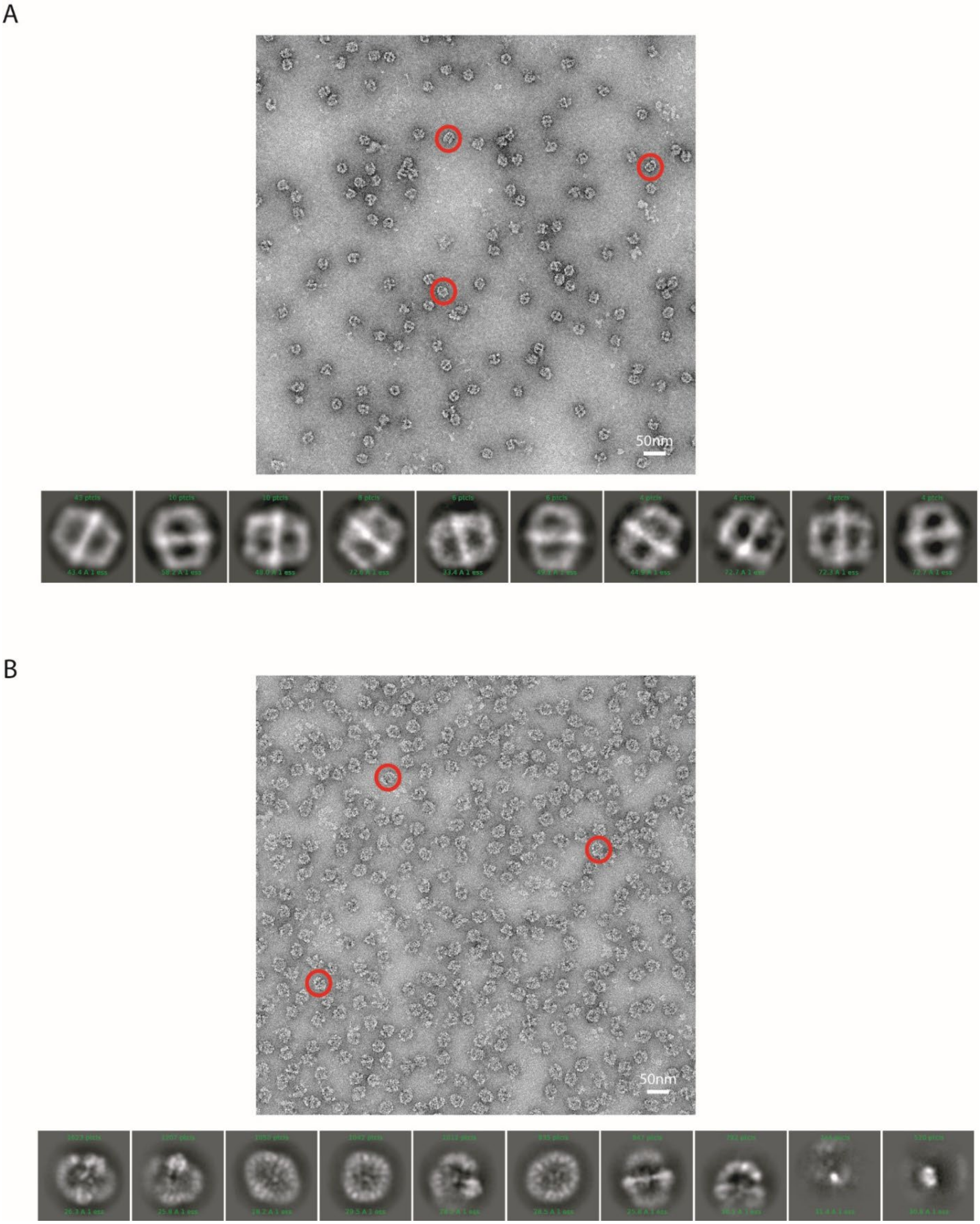

Supplementary figure 1. Insertion of six MBP tag on S. cerevisiae FAS destabilizes the structure. Representative negative stained electron micrographs and reference free 2D classes are shown for **A)** ^{fus}FAS and **B)** ^{fus}FAS$^{N\text{-}MBP}$ (*i.e.,* prep **2**).

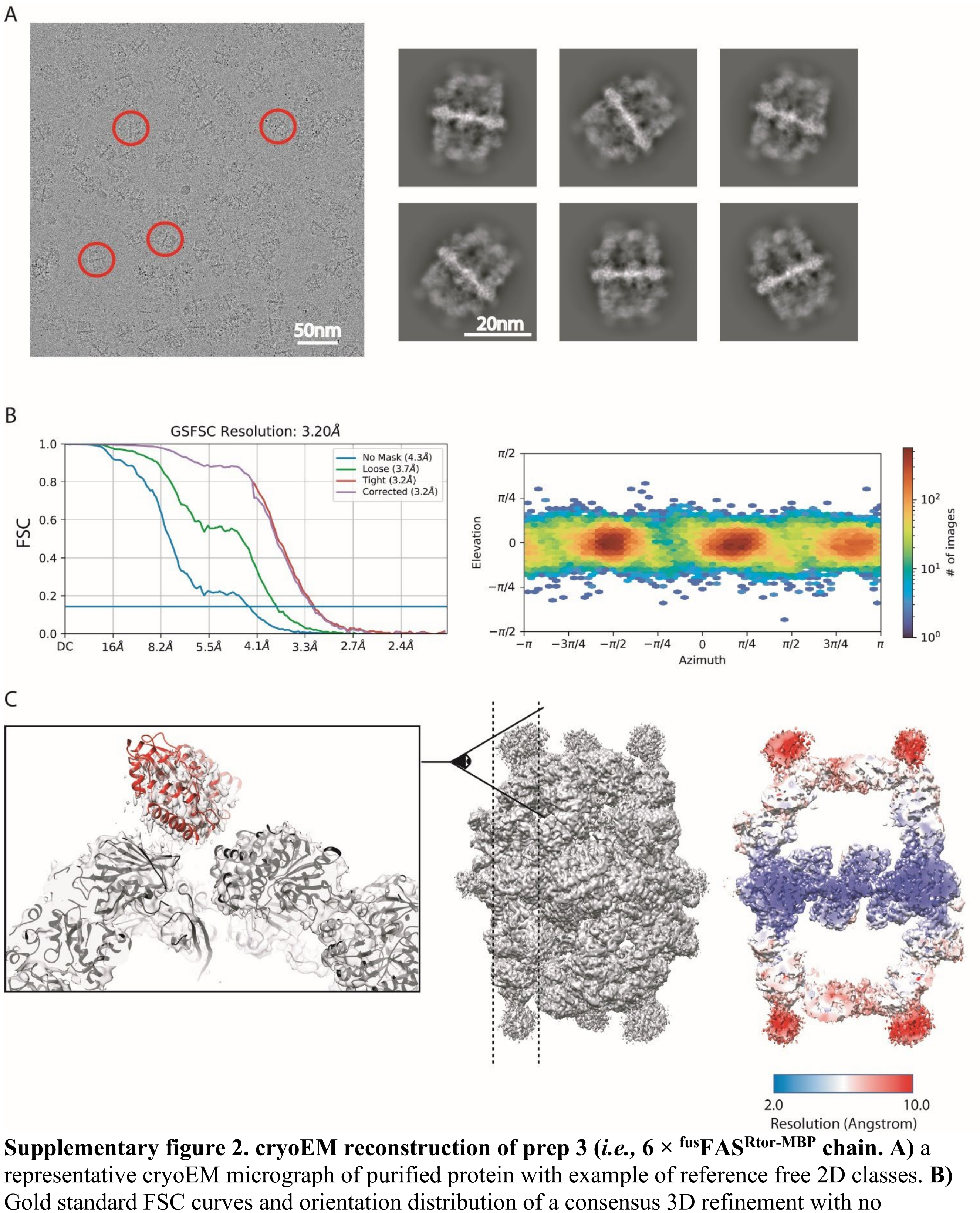

Supplementary figure 2. cryoEM reconstruction of prep 3 (*i.e.*, 6 × ^{fus}FAS$^{Rtor\text{-}MBP}$ chain. A) a representative cryoEM micrograph of purified protein with example of reference free 2D classes. **B)** Gold standard FSC curves and orientation distribution of a consensus 3D refinement with no symmetry imposed. **C)** cryoEM 3D reconstruction is shown in the center with model to map fit of the apical region shown in the inset. A slice of local resolution estimate is shown to the right.

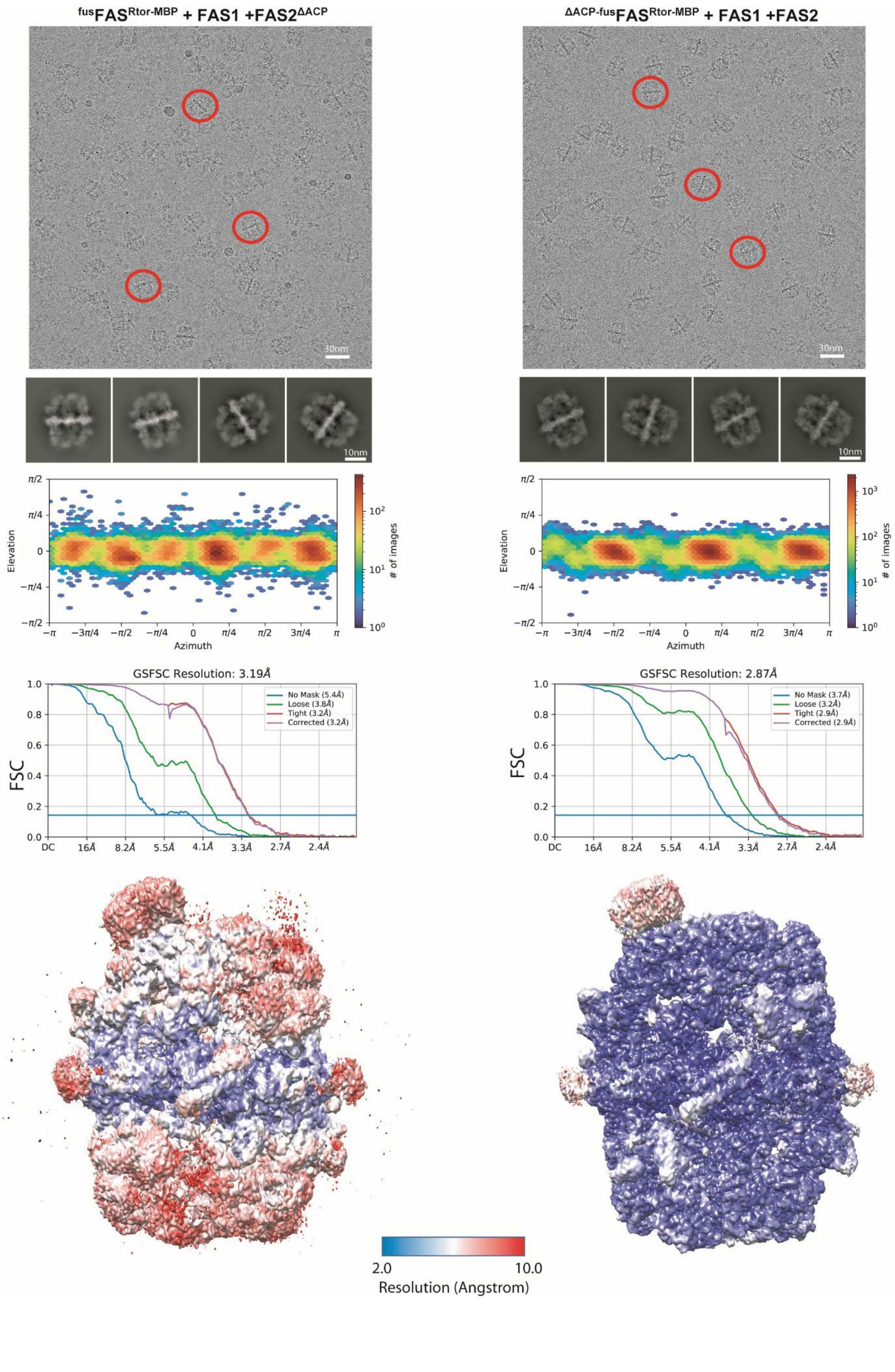
fusFASRtor-MBP + FAS1 +FAS2ΔACP
ΔACP-fusFASRtor-MBP + FAS1 +FAS2
30nm
30nm
10nm
10nm
Elevation
Azimuth
# of images
GSFSC Resolution: 3.19Å
No Mask (5.4Å)
Loose (3.8Å)
Tight (3.2Å)
Corrected (3.2Å)
GSFSC Resolution: 2.87Å
No Mask (3.7Å)
Loose (3.2Å)
Tight (2.9Å)
Corrected (2.9Å)
FSC
DC
16Å
8.2Å
5.5Å
4.1Å
3.3Å
2.7Å
2.4Å
2.0
10.0
Resolution (Angstrom)

Supplementary Figure 3. cryoEM analysis of asymmetric FAS. Representative cryoEM micrographs, reference free 2D classes, FSC curves, particle image orientation distribution, and 3D reconstructions colored via local resolution estimate are shown for each asymmetric FAS sample. Prep **4** and **5** are presented in the left and right columns, respectively.

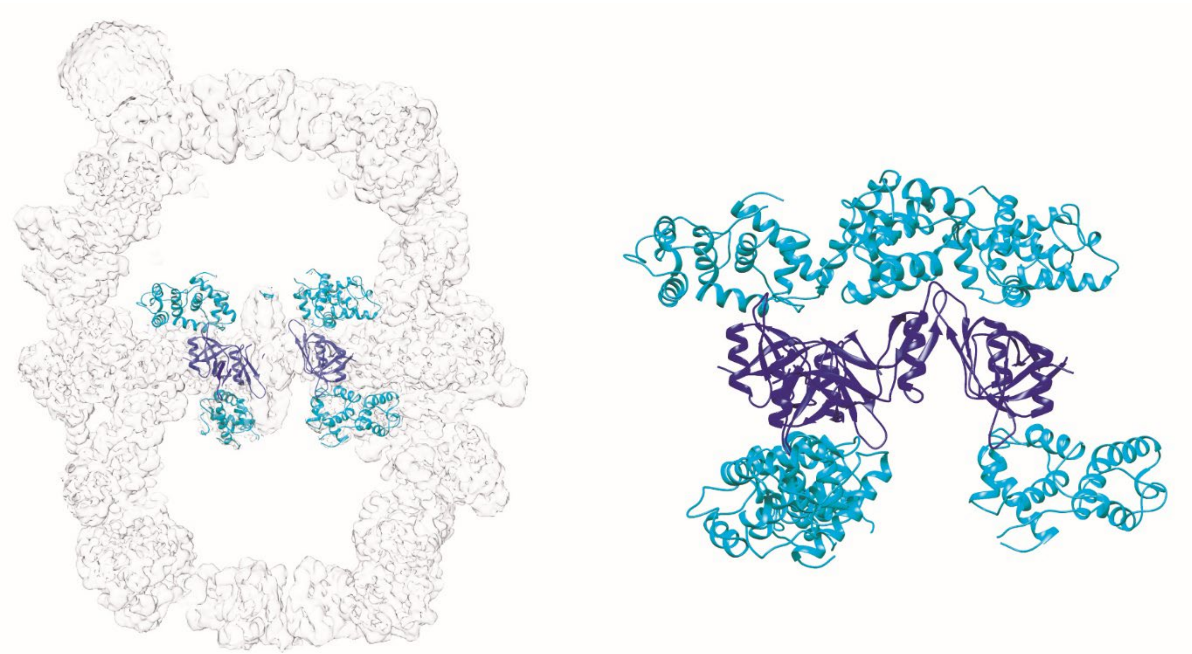

Supplementary figure 4. cryoEM density quantification of atomic models of ACP and KS domains. Atomic models of ACP domains (residues 141-302) and portions of KS domains (residue 1120-1179) are shown as cyan and dark blue respectively, fitted into the cryoEM map of asymmetric FAS from prep **5** shown as transparent white. Same fitting was done for cryoEM map of asymmetric FAS from prep **4**. Zoomed in view of atomic models used in quantification is shown in the right panel.

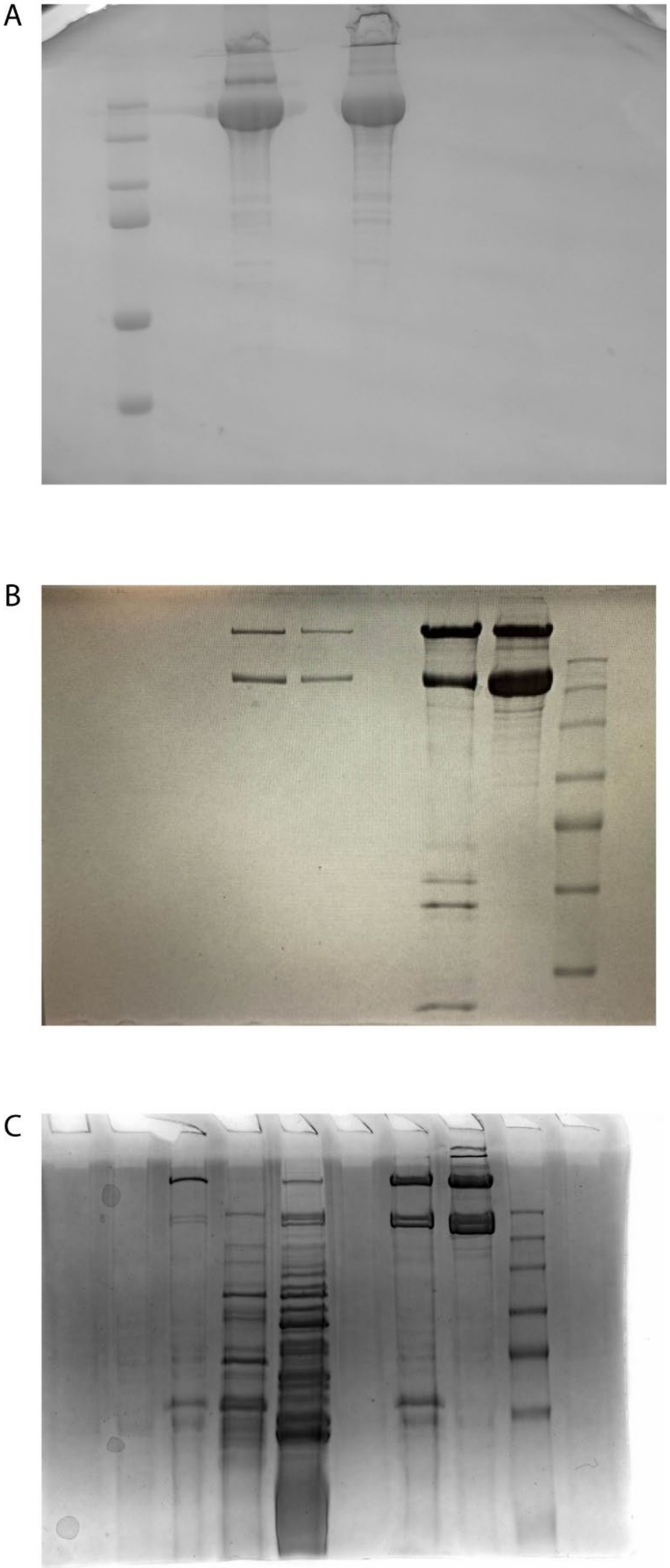

Supplementary figure 5. SDS-PAGE analysis of purified proteins. Uncropped and unedited images of SDS-PAGE analysis of proteins in this study. Panels **A)**, **B)**, and **C)** are from figure 2B, 3C, and 3D, respectively.

Supplementary References:

1. Wernig, F., Born, S., Boles, E., Grininger, M. & Oreb, M. Fusing α and β subunits of the fungal fatty acid synthase leads to improved production of fatty acids. *Sci Rep* **10**, (2020).

2. Lou, J. W., Iyer, K. R., Hasan, S. M. N., Cowen, L. E. & Mazhab-Jafari, M. T. Electron cryomicroscopy observation of acyl carrier protein translocation in type I fungal fatty acid synthase. *Sci Rep* **9**, 12987 (2019).

3. Gajewski, J., Pavlovic, R., Fischer, M., Boles, E. & Grininger, M. ARTICLE Engineering fungal de novo fatty acid synthesis for short chain fatty acid production. (2017) doi:10.1038/ncomms14650.
