# Supplementary material for: Direct structural analysis of a single acyl carrier protein domain in fatty acid synthase from the fungus Saccharomyces cerevisiae
Source: Commun Biol. 2024 Jan 12;7:92. doi: 10.1038/s42003-024-05777-7 (PMC10786820; doi:10.1038/s42003-024-05777-7)
Supplement: Supplementary file 2 — Description of Additional Supplementary Files [file 42003_2024_5777_MOESM2_ESM.pdf]

Description of Additional Supplementary Files

File name: Supplementary Data 1
Description: The source data behind the box plot in figure 5A.

File name: Supplementary Data 2
Description: The source data behind the box plot in figure 5B.
