# Supplementary material for: Direct structural analysis of a single acyl carrier protein domain in fatty acid synthase from the fungus Saccharomyces cerevisiae
Source: Commun Biol. 2024 Jan 12;7:92. doi: 10.1038/s42003-024-05777-7 (PMC10786820; doi:10.1038/s42003-024-05777-7)
Supplement: Supplementary file 5 — Reporting Summary [file 42003_2024_5777_MOESM5_ESM.pdf]

nature portfolio

Corresponding author(s): Mohammad T. Mazhab-Jafari

Last updated by author(s): Dec 17, 2023

Reporting Summary

Nature Portfolio wishes to improve the reproducibility of the work that we publish. This form provides structure for consistency and transparency in reporting. For further information on Nature Portfolio policies, see our Editorial Policies and the Editorial Policy Checklist.

Statistics

For all statistical analyses, confirm that the following items are present in the figure legend, table legend, main text, or Methods section.

n/a	Confirmed	
☐	☒	The exact sample size (*n*) for each experimental group/condition, given as a discrete number and unit of measurement
☐	☒	A statement on whether measurements were taken from distinct samples or whether the same sample was measured repeatedly
☐	☒	The statistical test(s) used AND whether they are one- or two-sided<br>*Only common tests should be described solely by name; describe more complex techniques in the Methods section.*
☒	☐	A description of all covariates tested
☒	☐	A description of any assumptions or corrections, such as tests of normality and adjustment for multiple comparisons
☐	☒	A full description of the statistical parameters including central tendency (e.g. means) or other basic estimates (e.g. regression coefficient) AND variation (e.g. standard deviation) or associated estimates of uncertainty (e.g. confidence intervals)
☒	☐	For null hypothesis testing, the test statistic (e.g. *F*, *t*, *r*) with confidence intervals, effect sizes, degrees of freedom and *P* value noted<br>*Give P values as exact values whenever suitable.*
☐	☒	For Bayesian analysis, information on the choice of priors and Markov chain Monte Carlo settings
☒	☐	For hierarchical and complex designs, identification of the appropriate level for tests and full reporting of outcomes
☒	☐	Estimates of effect sizes (e.g. Cohen's *d*, Pearson's *r*), indicating how they were calculated

Our web collection on statistics for biologists contains articles on many of the points above.

Software and code

Policy information about availability of computer code

Data collection	EPU v3.3.1.5184REL
Data analysis	cryoSPARC V3, UCSF Chimera v1.16

For manuscripts utilizing custom algorithms or software that are central to the research but not yet described in published literature, software must be made available to editors and reviewers. We strongly encourage code deposition in a community repository (e.g. GitHub). See the Nature Portfolio guidelines for submitting code & software for further information.

Data

Policy information about availability of data

All manuscripts must include a data availability statement. This statement should provide the following information, where applicable:

- Accession codes, unique identifiers, or web links for publicly available datasets
- A description of any restrictions on data availability
- For clinical datasets or third party data, please ensure that the statement adheres to our policy

All data and materials are available upon request. cryoEM density maps have been deposited in the Electron Microscopy Data Bank under accession codes: EMD-40783, EMD-40784, and EMD-40785.

Research involving human participants, their data, or biological material

Policy information about studies with human participants or human data. See also policy information about sex, gender (identity/presentation), and sexual orientation and race, ethnicity and racism.

Reporting on sex and gender	This is a structural biology study of a protein complex that is expressed in all humans. There are no sex and/or gender considerations
Reporting on race, ethnicity, or other socially relevant groupings	There are no human research participants in this study.
Population characteristics	There are no human research participants in this study.
Recruitment	There are no human research participants in this study.
Ethics oversight	Not applicable

Note that full information on the approval of the study protocol must also be provided in the manuscript.

Field-specific reporting

Please select the one below that is the best fit for your research. If you are not sure, read the appropriate sections before making your selection.

☒ Life sciences ☐ Behavioural & social sciences ☐ Ecological, evolutionary & environmental sciences

For a reference copy of the document with all sections, see nature.com/documents/nr-reporting-summary-flat.pdf

Life sciences study design

All studies must disclose on these points even when the disclosure is negative.

Sample size	The cryoEM sample size was determined by the ability to reach a global resolution of 3.5 Angstrom or better in the final 3D reconstructions.
Data exclusions	2D projections of contaminant and broken particles were excluded via 2D and 3D classification procedure as described. No other data exclusions were performed.
Replication	For cryoEM data, each dataset has hundreds of thousands to million particle images, therefore there is an inherent replication to the method. The large cryoEM dataset was not replicated due to cost of data collection on the electron microscope and the limitation of disk space.
Randomization	Samples (individual particle images) were allocated to experimental groups (i.e., 2D or 3D classes) based on the viewing direction and conformational variabilities. For resolution assessment of cryoEM maps, randomization was done by division into odd and even groups. No other randomization was performed.
Blinding	Blinding is not relevant to the cryoEM data analysis in this study since there are no placebo groups. Investigators in this study examined the grouping of cryoEM data since close examination of the cryoEM densities of each group classification was essential to identify the highest quality, highest resolution cryoEM 3D class.

Reporting for specific materials, systems and methods

We require information from authors about some types of materials, experimental systems and methods used in many studies. Here, indicate whether each material, system or method listed is relevant to your study. If you are not sure if a list item applies to your research, read the appropriate section before selecting a response.

Materials & experimental systems

n/a	Involved in the study	
☒	☐	Antibodies
☐	☒	Eukaryotic cell lines
☒	☐	Palaeontology and archaeology
☒	☐	Animals and other organisms
☒	☐	Clinical data
☒	☐	Dual use research of concern
☒	☐	Plants

Methods

n/a	Involved in the study	
☒	☐	ChIP-seq
☒	☐	Flow cytometry
☒	☐	MRI-based neuroimaging

Eukaryotic cell lines

Policy information about cell lines and Sex and Gender in Research

Cell line source(s)	Saccharomyces cerevisiae strains BY.PK1238 and W303
Authentication	Strains were authenticated by PCR from chromosomal DNA and purification of expected proteins from the yeast cells. Strains were also authenticated by growth on minimal media supplemented with specific nutritional markers and antibiotics that selected for genes on the chromosomal DNA.
Mycoplasma contamination	Not applicable to yeast cells.
Commonly misidentified lines (See ICLAC register)	Not applicable to yeast cells.
